# Unexplained diarrhoea in HIV-1 infected individuals

**DOI:** 10.1186/1471-2334-14-22

**Published:** 2014-01-13

**Authors:** Bas B Oude Munnink, Marta Canuti, Martin Deijs, Michel de Vries, Maarten F Jebbink, Sjoerd Rebers, Richard Molenkamp, Formijn J van Hemert, Kevin Chung, Matthew Cotten, Fransje Snijders, Cees JA Sol, Lia van der Hoek

**Affiliations:** 1 Laboratory of Experimental Virology, Department of Medical Microbiology, Center for Infection and Immunity Amsterdam (CINIMA), Academic Medical Center of the University of Amsterdam, Amsterdam, The Netherlands; 2 CBS-KNAW Fungal Biodiversity Center, Utrecht, The Netherlands; 3 Laboratory of Clinical Virology, Department of Medical Microbiology, Center for Infection and Immunity Amsterdam (CINIMA), Academic Medical Center of the University of Amsterdam, Amsterdam, The Netherlands; 4 Wellcome Trust Sanger Institute, Hinxton, CB10 1SA, UK; 5 Beatrix Hospital, Gorinchem, The Netherlands

**Keywords:** Diarrhoea, HIV-1, VIDISCA-454, Aichi virus, Human gyrovirus, Cosavirus, Immunodeficiency-associated stool virus, NonA-nonB hepatitis virus, Norovirus, Sapovirus

## Abstract

**Background:**

Gastrointestinal symptoms, in particular diarrhoea, are common in non-treated HIV-1 infected individuals. Although various enteric pathogens have been implicated, the aetiology of diarrhoea remains unexplained in a large proportion of HIV-1 infected patients. Our aim is to identify the cause of diarrhoea for patients that remain negative in routine diagnostics.

**Methods:**

In this study stool samples of 196 HIV-1 infected persons, including 29 persons with diarrhoea, were examined for enteropathogens and HIV-1. A search for unknown and unexpected viruses was performed using virus discovery cDNA-AFLP combined with Roche-454 sequencing (VIDISCA-454).

**Results:**

HIV-1 RNA was detected in stool of 19 patients with diarrhoea (66%) compared to 75 patients (45%) without diarrhoea. In 19 of the 29 diarrhoea cases a known enteropathogen could be identified (66%). Next to these known causative agents, a range of recently identified viruses was identified via VIDISCA-454: cosavirus, Aichi virus, human gyrovirus, and non-A non-B hepatitis virus. Moreover, a novel virus was detected which was named immunodeficiency-associated stool virus (IASvirus). However, PCR based screening for these viruses showed that none of these novel viruses was associated with diarrhoea. Notably, among the 34% enteropathogen-negative cases, HIV-1 RNA shedding in stool was more frequently observed (80%) compared to enteropathogen-positive cases (47%), indicating that HIV-1 itself is the most likely candidate to be involved in diarrhoea.

**Conclusion:**

Unexplained diarrhoea in HIV-1 infected patients is probably not caused by recently described or previously unknown pathogens, but it is more likely that HIV-1 itself plays a role in intestinal mucosal abnormalities which leads to diarrhoea.

## Background

One of the most affected sites in untreated human immunodeficiency virus (HIV)-infected persons is the gastrointestinal tract [1-5]. In these patients diarrhoea occurs frequently, especially in HIV-1 patients who are not treated with anti-retroviral therapy. Routine diagnostics in fecal samples often remain negative, despite the sensitive diagnostic assays which are now available. In this study our aim is to identify the cause of diarrhoea for patients in which no enteropathogen could be found. There are several possible explanations why these patients remain negative in routine diagnostics: First, HIV-1 itself might play a role. HIV-1 infected mucosal cells have been observed in HIV-1 infected persons [6-11] and enterocytes exposed to HIV-1 show altered function and proliferation [12,13]. However, it has not been demonstrated that HIV-1 causes diarrhoea. Secondly, there are several recent reports describing novel viral agents which are found in stool that could play a role in diarrhoea (see below), and these agents are not yet included in routine diagnostics. Thirdly, an unknown or unexpected virus might be causing diarrhoea.

One of the recently described gastrointestinal viruses is cosavirus, which was identified in 2008 as a member of the *Picornaviridae*[14]. The virus was originally found in 2 patients with gastrointestinal infections [14,15]. However, its link with diarrhoea has not been established and a recent study in HIV-infected individuals detected cosavirus in a substantial number of patients without diarrhoea [16]. Aichi virus is another recently described picornavirus which was identified in fecal samples of patients with gastroenteritis [17]. Several manuscripts have since then confirmed that the virus is associated with diarrhoea [18,19], although in one study, which included children without diarrhoea, the association with disease could not be established since the amount of positive samples was too low [20]. Human gyrovirus, a member of the *Circoviridae*, is the most unusual novel virus which is found in gastrointestinal infections [21]. The virus is genetically very closely related to gyroviruses found in chicken meat [22] and therefore it remains uncertain whether human gyrovirus in stool is a sign of gastrointestinal infection or a virus which is digested with food and shed in feces, like many food related viruses [23].

Identification of unknown and unexpected viruses is performed via virus discovery cDNA-AFLP followed by Roche 454 sequencing (VIDISCA-454) [24]. This technique, which can detect single stranded and double stranded RNA and DNA viruses [25], has been developed in our laboratory and is a sensitive next generation sequencing-based assay which enables detection of known and unknown viruses in a wide range of different sample types, including stool samples [24,26-28].

In the current study, a cohort of 196 HIV-1 infected individuals of whom 29 suffered from severe diarrhoea was investigated. In this patient group we determined how many cases of diarrhoea could be explained by known enteropathogens, recently described viruses and unknown viruses.

## Methods

### Patients

One hundred and ninety-six HIV-1 infected patients, who visited the out-patient clinic at the Amsterdam Medical Center in 1994 and 1995, not on active antiretroviral therapy, were asked to bring a fresh stool sample at their next visit [29]. Criteria for inclusion in the study were proven HIV-1 infection and being aged 18 years or older.

Patients were asked to complete a questionnaire about the frequency and consistency of their stools during the week before the stool was collected. The definition of diarrhoea was loose or watery stool at least 3 times a day during the week prior to faeces collection (in accordance with the WHO guidelines for diarrhoea). In addition, patients were asked for the occurrence of additional symptoms and use of medication that might affect the consistency of stool. Hospital charts of patients were reviewed for additional clinical data and the local medical ethical committee of the AMC approved the study. Demographical data of our study population are shown in Table 1. Cultures were performed less than 4 hours after the specimen was collected (see below). Blood for serum storage and blood for CD4 cell count measurements were collected at the same date as stool collection. Stool samples were suspended in broth (1:3 dilution, Oxoid nutrient broth no.2, pH 7.5 and 500 IU penicillin per ml, 3 μg amphotericin B per ml). Serum and stool suspensions were stored at −80°C until further use.

**Table 1:** Demographic data of the study population

	**All (n = 196)**	**Diarrhoea (n = 29)**	**No diarrhoea (n = 167)**
*Age, mean (SD)*	41 (7)	41 (7)	41 (7)
*Male*	93% (183)	97% (28)	93% (155)
*Female*	7% (13)	3% (1)	7% (12)
*CD4 count, mean (IQR)*^1^	0.17 (0.04-0.29)	0.11 (0.01-0.20)	0.18 (0.04-0.29)
*CDC stage*^2^			
1	22% (43)	9% (4)	91% (39)
2	29% (56)	13% (7)	88% (49)
3	49% (97)	19% (18)	81% (79)
*Risk group*			
Homosexual	85% (166)	17% (28)	83% (138)
Heterosexual	11% (21)	5% (1)	95% (20)
Haemophiliac	1% (2)	0% (0)	100% (2)
Intravenous drug use	4% (7)	0% (0)	100% (7)
Blood transfusion	1% (1)	0% (0)	100% (1)
*HIV-1 SI phenotype*	32% (62)	21% (13)	79% (49)
*P24 antigen in serum*	16% (32)	3% (1)	97% (31)
*HIV-1 RNA load >4.9 × 10*^*4*^*copies/ml*	51% (99)	19% (19)	81% (80)

^1^IQR = inter quartile range.

^2^Stage 1: symptom free, 2: AIDS related symptoms that were not AIDS defining, 3: AIDS defining illness according to the CDC classification [30].

### CD4 cell counts

T lymphocyte immunophenotyping for CD4 was carried out by flow cytofluorometry. PBMC were stained with CD4 mAb (Leu-3a-PE; Becton Dickinson, Mountain View, CA) according to the manufacturer’s protocol.

### Virus diagnostics

DNA was extracted from 110 μl stool suspensions by the Boom extraction method [31]. Reverse transcription was performed as described [32]. Real time (RT-)PCRs specific for rotavirus A and C, enterovirus, human parechovirus, norovirus genogroup I and II, astrovirus, sapovirus and adenovirus 40, 41 and 52 was performed (see Additional file 1: Table S1 for primer sequences) [32]. As internal control Equine Arteritis Virus (EAV) RNA was included. HIV-1 RNA detection in stool was determined as described [33]. Screening for viruses which were not in the diagnostic panel was done by PCR. Gyrovirus, Aichi virus, and non-A non-B hepatitis virus (NANBH virus) were diagnosed via nested PCR (see Additional file 1: Table S1 for primer sequences). All positive signals were confirmed by Sanger sequencing.

### Stool examination for intestinal parasites

Intestinal parasites were detected in fresh stool by light microscopy: examination of direct smears and the Ridley concentration technique were both performed. Acid-fast staining was performed for detection of *Cryptosporidia* and *Isospora* species. Fluorescent staining with Uvitex 2B (Ciba-Geigy, Basel, Switzerland) was performed for detection of *Microsporidia*[34].

### Culture of enteropathogenic bacteria

To isolate *Salmonella* and *Shigella* species, fresh stool specimens (within 4 hours after specimen collection) were plated on cystine lactose electrolyte-deficient medium (Brocalin agar; Merck, Darmstadt, Germany), *Salmonella*-*Shigella* agar (Merck), Hektoen enteric agar (Difco, Detroit), Rappaport medium (Difco), MacConkey agar (Becton Dickinson, Cock-eysville, MD) and gram-negative broth (Hajna; Difco) as enrichment. A selective cefsulodin-irgasan-novobiocin medium (Oxoid, Basingstoke, Hampshire, England) and a glucose broth enrichment medium were used to isolate *Yersinia* species. Isolates were further characterized by using conventional bacteriologic methods, as outlined [35]. A cell-culture assay was used to detect *Clostridium difficile* toxin [36]. The *Campylobacter*-selective medium (modified Butzler medium) consisted of a solid 7% hemolyzed sheep blood–based agar (nutrient broth no. 2) with cefoperazone (30 mg/L), rifampin (10 mg/L) and seroamphotericin B (2 mg/L) [29].

### VIDISCA and Roche Titanium-454 sequencing

VIDISCA-454 was performed on 110 μl stool suspension as previously described by De Vries et al. [25]. VIDISCA-amplified nucleic acid - each with its own multiplex identifier (MID) - was quantified with the Quant-it dsDNA HS Qubit kit (Invitrogen) and diluted to 10^6^ copies/ml. All separate fractions were pooled and 4 pools from 14 different samples were used for a titration (DNA:beads ratio of 0.5:1, 1:1, 2:1 and 4:1) in an emulsion PCR according to the suppliers’ protocol (LIB-A SV emPCR kit). Sequencing was done on a 4 region GS FLX Titanium PicoTiterPlate (70x75) with the GS FLX Titanium XLR 70 Sequencing kit (Roche).

### Sequence analysis

Primer, MID and ribosomal RNA sequences were trimmed or removed from the reads. Sequences were assembled with CodonCode Aligner software version 3.5.6. The consensus sequences (contigs) and the unassembled reads were compared with the available sequences in Genbank [37] via the BlastN tool using default settings [38]. The blast output was used to create a taxonomic classification of the reads with Megan software version 4.70.4 [39]. The following settings were used: Min Support: 1, Min Score: 75, Min Score/Length: 0.75, Top Percent 100. Sequences without homology to known sequences were compared with less stringent settings to the available sequences in Genbank (expect threshold: 1000, Match/Mismatch Scores: 1.-1, Gap Costs: Existence: 2 Extension: 1), allowing more mismatches to occur in the comparison. Subsequently, the taxonomic classification of reads was also made with less stringent setting (Min Support: 1, Min Score: 10, Min Score/Length: 0.00, Top Percent 100). Additional file 1: Figure S1 represents a schematic overview of our analysis method.

### Statistical analysis

Statistical analysis to determine the association with diarrhoea was performed with the Mid-P exact test, using the two by two table from OpenEpi [40]. Statistical analysis for the CD4 cell counts and the HIV-1 load was performed with the test of equality variance from OpenEpi [40].

### Accession numbers

The complete genome sequence of NANBH-1 and NANBH-2 (uncultured phage WW-nAnB strain 2 and 3) and IAS virus have been deposited in GenBank under the accession numbers KJ003981 – KJ003983.

## Results

### Known enteropathogens

To investigate how many diarrhoea cases of HIV-1 infected individuals could be explained by infections with known pathogens, real time PCR, bacteria culture and microscopy was performed on stool samples of 196 HIV-1 infected patients of whom 29 had severe diarrhoea. The known diarrhoea agents parechovirus, astrovirus, rotavirus or adenovirus 40, 41 or 52 were not found in any of the 196 HIV-infected patients (see Table 2). Sapovirus was found in 3 patients with diarrhoea (10%) and once in the control group (patients without diarrhoea), whereas norovirus was present in 10 patients with diarrhoea (34%) compared to 13 controls (7.8%). Statistical analysis showed that norovirus as well as sapovirus are associated with diarrhoea (p value: 0.0006 and 0.02 respectively). Enterovirus was present in 3 patients with diarrhoea (10%) and in 5 patients in the control group (3.0%), which is statistically not significantly different.

**Table 2:** Prevalence of known enteropathogens

	**Diarrhoea (n = 29)**	**Non diarrhoea (n = 167)**	P-value*
Norovirus	34% (10)	7.8% (13)	*0.0006*
Astrovirus	0% (0)	0% (0)	-
Adenovirus	0% (0)	0% (0)	-
Sapovirus	10% (3)	0.6% (1)	*0.02*
Enterovirus	10% (3)	3.0% (5)	0.20
Rotavirus	0% (0)	0% (0)	-
Parechovirus	0% (0)	0% (0)	-
HIV-1 in stool	66% (19)	45% (75)	0.06
*Mycobacterium*	3.4% (1)	0% (0)	0.29
*Salmonella*	3.4% (1)	0% (0)	0.29
*Shigella*	0% (0)	0% (0)	-
*Yersinia*	0% (0)	0% (0)	-
*Clostridium diff*	0% (0)	0.6% (1)	0.99
*Campylobacter*	10% (3)	3.0% (5)	0.20
*Microsporidia*	6.9% (2)	4.2% (7)	0.79
*Giardia lamblia*	3.4% (1)	0.6% (1)	0.55
*Cryptosporidia*	10% (3)	0.6% (1)	*0.02*
Pathogen negative	34% (10)	83% (139)	

*P-values (Mid-P extract) in italics represent significant p-values for associating with diarrhoea.

Five patients with diarrhoea (16.8%) were infected with bacteria: *Salmonella* n = 1, *Mycobacterium* n = 1 and C*ampylobacter* n = 3. On the other hand, bacteria were also observed in 6 patients (3.6%) without diarrhoea (C*lostridium difficile* n = 1 and C*ampylobacter* n = 5). *Shigella* and *Yersinia* were not detected in patients with or without diarrhoea. None of the bacterial infections were significantly associated with diarrhoea. Parasites were found in 6 patients (21%) with diarrhoea (*Cryptosporidia* n = 3, *Giardia lamblia* n = 1 and *Microsporidia* n = 2), and in 9 patients (5.4%) of the control group without diarrhoea (*Cryptosporidia* n = 1, *Giardia lamblia* n = 1 and *Microsporidia* n = 7). Of all parasitic infections, only *Cryptosporidia* was significantly associated with diarrhoea (p = 0.02). We also screened for *Cyclospora*, *Isospora*, worm eggs and cysts but these pathogens were not significantly associated with diarrhoea in our study population (data not shown).

### VIDISCA-454

After screening for known viruses, bacteria and parasites, 10 patients with diarrhoea (34%) remained without diagnosis. We hypothesize that these patients are infected by an unknown or unexpected viral pathogen, and therefore a search for unknown or unexpected viruses was performed. A total of 56 stool samples, including samples that remained negative in diagnostics, were selected and investigated by VIDISCA-454. Approximately 5,000 sequences per sample were compared with all sequences in the non-redundant database of Genbank, using the BLAST tool from NCBI (see Additional file 1: Figure S1). A total of 7,316 known viral sequences could be identified this way (2.7% of all sequences, see Table 3).

**Table 3:** Quantification of the sequence reads from virus in stool samples of HIV-1 infected individuals

**Virus:**	**Amount of sequences:**
Bacteriophages	4269
IAS virus	2201
Plant viruses	1115
Cryptosporidium parvum-virus	813
Torque teno virus	662
Norovirus	215
Sapovirus	90
Adenovirus D	84
Enterovirus	37
Cosavirus	11
NANBH-1 virus	8
JC polyomavirus	4
Human papillomavirus	4
Aichi virus	3
Hepatitis B virus	3
Picobirnavirus	3
Gyrovirus	2
Rhinovirus A	1

Several viruses known to cause diarrhoea were found: sapovirus, enterovirus and norovirus (see Table 3). Furthermore, new candidate diarrhoea viruses were detected: human gyrovirus, cosavirus, and Aichi virus. In addition, known viruses of which it is not likely that they are involved in diarrhoea were detected: human adenovirus D viruses (type 23, 45 and 51, types which are not associated with diarrhoea), JC polyoma virus (probably originating from urine [41]), hepatitis B virus, rhinovirus (probably originating from respiratory tract) and human papilloma viruses. Furthermore torque teno viruses (TTV) and picobirnaviruses were found. TTVs represent an extremely diverse set of viruses which can differ substantially in their genome sequence and the size of their genome. These viruses have been detected in a large variety of clinical samples, and their association with any disease has not been established thus far [42]. Also picobirnaviruses are exceptionally diverse, they have been detected in diarrhoea patients, however, during outbreaks a diverse swarm of picobirnaviruses is observed making it less likely that these viruses cause diarrhoea [43].

Interestingly, a high amount of sequences of Cryptosporidium parvum-virus was found in a patient infected with *Cryptosporidium parvum*. Detection of this virus in stool of a patient never has been reported, although it has been suggested to perform screening for the presence of this virus in environmental samples to determine if water is infected with the parasite [44].

Two variants of non-A non-B hepatitis virus (NANBH-1 and NANBH-2) were found. The NANBH virus has been described in the 1980’s in patients with non A non B hepatitis, at a time that hepatitis C virus was not identified yet [45]. Since then no other study has investigated its presence in humans. NANBH viruses are circular dsDNA viruses and since the viruses were found by VIDISCA-454 in two patients with unexplained diarrhoea, their entire genome was sequenced. The viral genomes consist of 5,005 (NANBH-1) and 5,687 (NANBH-2) bps and both contain 4 open reading frames. The NANBH-2 virus is similar to the NANBH-1 except for a 687 nucleotide insertion in a non-coding region.

In addition, one novel virus was identified among the VIDISCA-454 reads, which we named immunodefiency-associated stool virus (IAS virus). The virus was present in a high concentration in stool since large numbers of sequence reads belonged to the virus (2201 of 7316 sequences, see Table 3 and Additional file 1: Table S2). The complete genome sequence of IAS virus was determined. This novel virus carries a dsDNA genome of 99.980 bp. The virus is exceptional because it displays no significant similarity to any of the viruses known thus far. Real time PCR confirmed that the virus is present at a high load in the sample in which the virus was discovered (1 × 10^7^ DNA copies/ml).

In addition to mammalian or vertebrate viruses, several bacteriophages and plant viruses were found. A total of 1,115 plant virus sequences (0.4%) and 4,269 bacteriophage sequences (1.6%) were identified (see Table 3 and Additional file 1: Table S2).

### Screening for novel and unexpected viruses

Among the viruses which were detected via VIDISCA-454, there are several candidates that might be involved in the patients’ diarrhoea. We focused on the most likely candidates to be involved in intestinal malfunctioning: human gyrovirus, cryptosporidium parvum-virus, Aichi virus and cosavirus, together with the new IAS virus and the unexpected NANBH virus. For these viruses a diagnostic PCR assay was developed (see Additional file 1: Table S1 for primer sequences). Of note, TTV and picobirnavirus, found by VIDISCA-454, were not included in the screening. Both virus families are extremely diverse and it is difficult, if not impossible, to develop proper diagnostic primers that can detect all variants. Furthermore, in literature TTVs have not been related to gastrointestinal disease.

All 196 patients - diarrhoea patients and subjects without diarrhoea - were tested for the abovementioned viruses. Cosavirus was found in 1 patient (3.4%) with and in 2 patients (1.2%) without diarrhoea (see Table 4). Human gyrovirus was detected in 33 individuals (17%): in 29 controls (17%) and 4 diarrhoea patients (14%). Also Aichi virus was frequently found: 40 individuals (20%) were positive of whom 34 had no diarrhoea (20%) and 6 diarrhoea (21%). IAS virus was detected in 2 patients with diarrhoea (6.9%) and in 6 individuals without diarrhoea (3.6%). The NANBH virus was detected in 2 patients with diarrhoea (6.8%) and in 14 patients without diarrhoea (8.4%). Thus, none of the novel or recently described viruses could be associated with diarrhoea.

**Table 4:** Prevalence of new viruses

	**Diarrhoea (n = 29)**	**Non diarrhoea (n = 167)**	**P-value**
IAS virus	6.9% (2)	3.6% (6)	0.67
NANBH virus	6.8% (2)	8.4% (14)	0.99
Cosavirus	3.4% (1)	1.2% (2)	0.77
Cryptosporidium parvum-virus	6.9% (2)	1.8% (3)	0.32
Human gyrovirus	14% (4)	17% (29)	0.87
Aichi virus	21% (6)	20% (34)	0.99

Intriguingly, detection of the IAS virus was associated with low CD4 cell counts (p < 0.01, two-tailed t-test), and exclusively occurred in patients with CDC-stage 2 or 3. In contrast, detection of NANBH virus and human gyrovirus presence was not related to CD4 cell counts, and also occurred in the CDC-stage 1 patients (data not shown). However, Aichi virus detection was strongly related to CDC stage of the patient (P < 0.0001) with most patients in CDC stage 2 or 3 being positive, however no relation with CD4 cell counts was observed.

*Cryptosporidium parvum* is a parasite which causes diarrhoea and screening for the virus which infects this parasite can potentially be used as an additional marker, next to the microscopic analysis for identification of the parasite. Microscope analysis showed that 3 patients with diarrhoea (10%) and 1 patient without diarrhoea (0.6%) were infected with *Cryptosporidium parvum*. The virus infecting the parasite was found in 2 patients with diarrhoea (6.9%) and in 3 patients without diarrhoea (1.8%) and was not associated with diarrhoea. Only one sample was found positive in both assays.

The role of HIV-1 itself in the unexplained diarrhoea was also examined. HIV-1 RNA was detectable in stool of 19 patients with diarrhoea (66%) compared to 75 patients without diarrhoea (45%). This association was not significant (p value: 0.06). It could be that HIV-1 shedding is just a sign of infection by pathogens, and that shedding of the virus occurs in case an enteropathogen disrupts the mucosal barrier. However, unexpectedly, in the group without enteropathogens the frequency of HIV-1 RNA detection in stool was higher (8 out of 10, 80%) compared to the group where an enteropathogen could be found (9 out of 19, 47%), thus even an inverse relationship between HIV-1 shedding and infection by known pathogens was measured.

Finally, we could exclude that the use of antibiotics or other treatment was the cause of diarrhoea. In total 91 patients were treated with antibiotics, most of them with a combination of sulfamethoxazole and trimethoprim and there was no difference in antibiotic usage in diarrhoea cases compared to the control patients without diarrhoea (41% versus 49%).

## Discussion

Since the gastrointestinal tract is one of the most affected sites in untreated HIV-1 infections, the cause of diarrhoea in HIV-1 infected individuals was sought. After testing for all known enteropathogens, a pathogenic agent was detected in 66% of the diarrhoea cases. Virus infection accounted for 54% of the diarrhoea cases, bacterial infection was identified in 17%, parasitic infection in 21% and also some dual infections were observed. Of all viruses, norovirus accounts for the majority of infections (34%), followed by sapovirus and enterovirus (10% each). Still, in 34% of the patients with diarrhoea the causative agent could not be found. A careful investigation of the cause of unexplained diarrhoea in these 10 patients was performed using deep sequencing strategies. We were able to identify several recently described and one new virus with VIDISCA-454 (e.g. NANBH virus, cosavirus, Aichi virus, human gyrovirus and a novel IAS virus). However, no association between these viruses and diarrhoea was observed.

Remarkably, the prevalence of Aichi virus in our study population was 20%, while previous studies only showed a prevalence of 0.5-1.6% [19,46]. It could be that the virus is more frequently present in patients with immunodeficiency. Furthermore, the sensitivity of our assay may also play a part. In our study a very sensitive nested PCR assay was used which enables detection of the virus even if it is present at low concentrations. To exclude that the high prevalence was caused by PCR contamination, all positive PCR products were sequenced. Variation between the viruses from different patients was observed, thereby excluding contamination by a single source (see nucleotide alignments in Additional file 1: Table S3).

Human gyrovirus was detected in 33 patients. This study is the first study that shows that this virus was already present in the Netherlands in 1994 and 1995. The virus was present in 17% of the individuals without diarrhoea, compared to 14% of the patients, indicating that it is unlikely that the virus is involved in disease. Since antibody response against this virus has not been described thus far, combined with the fact that this virus was also found in chicken [22], we hypothesize that detection of human gyrovirus in stool may very well be food related.

Cryptosporidium parvum-virus PCR screening resulted in 5 positive samples, but it also showed that detection of the virus is not associated with diarrhoea. Screening for cryptosporidium parvum-virus has already been suggested as an alternative test to detect the parasite in surface water [44]. However, since microscopic analysis for the parasite itself did show a diarrhoea-association, we propose that only screening for the *Cryptosporidium parvum*, and not the virus infecting the parasite, should be performed in diagnostics of patients.

The NANBH virus was present in some of our study subjects in high viral loads. Recently, an Inovirus (a bacteria infecting virus family) was described by Cantalupo et al. [47] that resembles the NANBH virus (90% identity). This resemblance strongly indicates that the NANBH-1 and the NANBH-2 viruses are bacteriophages and in this respect it is not surprising that no correlation with diarrhoea was found here.

Finally, the role of HIV-1 self was investigated. Remarkably, although not significant, HIV-1 RNA was detected more in stool samples in patients where no enteropathogen could be found compared to the group with enteropathogen. This result indicates that shedding of HIV-1 in feces is not the direct result of damage to the mucosal barrier by enteropathogens, but that HIV-1 itself may damage the mucosal barrier and cause diarrhoea. Detection of HIV-1 in stool was not related to the stage of HIV-1 disease since CD4 cell count, CDC stage, and HIV-1 viral load did not differ significantly between diarrhoea patients with enteropathogen and people without enteropathogen (data not shown). The relatively low number of patients with unexplained diarrhoea is a limitation of our study. In the future, a study focusing on unexplained diarrhoea should include more HIV-1 positive patients with unexplained diarrhoea to be able to confirm our hypothesis that HIV-1 itself plays a role in gastrointestinal disorders.

## Conclusions

Virus discovery has in recent years revealed several previously unknown viruses for which it has been postulated that they are involved in diarrhoea. In the study presented here we show that infection by these novel agents does not explain the high frequency of diarrhoea in HIV-1 infected patients. Also the novel IAS virus could not be linked to diarrhoea, but interestingly, it was associated with advanced stages of HIV-1 disease. Further investigation of this virus may reveal whether the virus is a human virus or a virus infecting bacteria. Sequence comparison could not clarify this, since the virus has no sequence identity to any of the viruses known thus far.

In the early HIV-1 literature it has been shown that HIV-1 has an effect on enterocytes. This knowledge combined with our detection of HIV-1 in stool - especially in those patients which remained negative in diagnostics – suggests that HIV-1 may cause the gastrointestinal damage and the resulting diarrhoea. Further research with cell culture based techniques is needed to reveal the role of HIV-1 in gastrointestinal disease.

## Competing interests

The authors declare that they have no competing interests.

## Authors’ contributions

BBOM carried out the virus discovery studies and drafted the manuscript. MCa, MD, and MdV carried out the VIDISCA-454 runs, MFJ carried out sequence analysis, SR and RM performed the viral diagnostics, FJvH carried out protein structure analysis, MCo and KC participated in full length sequencing of unknown viruses, FS was involved in design of the study and patient inclusion, CJAS initiated the study, LvdH supervised the study, participated in its design, and helped to draft the manuscript. All authors except CJAS have read and approved the final manuscript.

## Pre-publication history

The pre-publication history for this paper can be accessed here:

http://www.biomedcentral.com/1471-2334/14/22/prepub

## Supplementary Material

Additional file 1: Table S1 Primer combination used for the screening. **Table S2**: Quantification of the detection of viral families in stool samples of HIV-1 infected individuals. **Table S3**: Alignment of the sequences of the different aichi viruses. **Figure S1**: Pipeline of the data analysis. Click here for file
