# Supplementary material for: Unexplained diarrhoea in HIV-1 infected individuals
Source: BMC Infect Dis. 2014 Jan 13;14:22. doi: 10.1186/1471-2334-14-22 (PMC3925291; doi:10.1186/1471-2334-14-22)

**Additional file:**

**Table S**1: Primer combination used for the screening

| Adeno 40/41 | F | TTCCAGCATAATAACTCWGGCTTTG |
| --- | --- | --- |
|  | R | AATTTTTCTGWGTCAGGCTTGG |
|  | P | CCWTA+CCC+C+CTTATT+GG* |
| Adeno52 | F | AACAGATACCGCAACCACCC |
|  | R | CCTGCCACTTTATCATTAGTGCCTA |
|  | P | TATCAA+CCT+GAA+C+CA+CAAG |
| Sapo | F | GACCAGGCTCTCGCYACCTAC |
|  | R | CCCTCCATYTCAAACACTAWTTTG |
|  | P | TGGTT+CATA+G+GT+G+GTAC |
| Rota A | F | ACCATCTWCACRTRACCCTC |
|  | R | GGTCACATAACGCCCC |
|  | P | ATGAGCACAATAGTTAAAAGCTAACACTG+T+CAA |
| Rota C | F | CTACAAGTAATGGAATCGGATG |
|  | R | TGGGTGTCATTTGATACAACTTCA |
|  | P | ACCAGCTAGTA+C+A+G+A+AAC |
| Noro-G1 | F | ATGTTCCGCTGGATGCG |
|  | R | CGTCCTTAGACGCCATCATC |
|  | P | TGGACAGGAGATCGC |
| Noro-G2 | F | CAAGAICCIATGTTYAGITGGATGAG |
|  | R | TCGACGCCATCTTCATTCAC |
|  | P | TGGGAGGGCGATCG |
| Astro | F | GACTGCWAAGCAGCTTCGT |
|  | R | GCCATCACACTTCTTTGGT |
|  | P | TCACAGAAGAGCAACTCCATCGCATTTG |
| EAV | F | CATCTCTTGCTTTGCTCCTTAG |
|  | R | AGCCGCACCTTCACATTG |
|  | P | TGTGGGCAATAATGTTGTTCTGACAGCG |
| Entero | F | GGCCCTGAATGCGGCTAAT |
|  | R | GGGATTGTCACCATAAGCAGCC |
|  | P | GCGGAACCGACTACTTTGGGT |
| HPeV | F | CTGGGGCCAAAAGCCA |
|  | R | GGTACCTTCTGGGCATCCTTC |
|  | P | AAACACTAGTTGTAWGGCCC |
| Aichi | F | GGCCACACAACCAGAGGGATTCC |
|  | R | GGAGGTCGCTACATCCACTCCATC |
| Cosa | F | GACTCTGTCTTCCATGTTTGTGTCC |
|  | R | GCTTCTATTGATGCTTCTGCCTCC |
| IAS virus | F | GTCTACCATGGCAACCAATCCT |
|  | R | GCCRAGAAGACCAGAAGCAC |
| NANBH-1 virus | F | CACCTCCATCCCAAAGGCCAC |
|  | R | GCCRAGAAGACCAGAAGCAC |
| NANBH-1 virus nested | F | CACCTCCATCCCAAAGGCCAC |
|  | R | GCCRAGAAGACCAGAAGCAC |
| Gyrovirus | F | ATGTCATCCGGCGGTCTCGG |
|  | R | CACTCACTCGGTGGTACGCTG |
| Gyrovirus nested | F | CGCCAGAGATCTACGTCGGC |
|  | R | GGCTTCTACCACAGAGGACGAT |
| Cryptosporidium parvum virus | F | GCCCAGATTAGACGAGGTTTATTCTC |
|  | R | GATGCGTCTGGCTCGATCTCAATGG |

* For all probes, N+ denotes a Locked Nucleic Acid nucleotide.

**Table S2: Quantification of the detection of viral families in stool samples of HIV-1 infected individuals.**

| Siphoviridae | 3526 |
| --- | --- |
| Myoviridae | 853 |
| Virgaviridae | 759 |
| Anelloviridae | 557 |
| Partitiviridae | 466 |
| Tombusviridae | 344 |
| Microviridae | 311 |
| Caliciviridae | 283 |
| Adenoviridae | 87 |
| Podoviridae | 58 |
| Picornaviridae | 48 |
| unclassified phages | 32 |
| Mimiviridae | 10 |
| Bromoviridae | 5 |
| Inoviridae | 4 |
| Papillomaviridae | 4 |
| Polyomaviridae | 4 |
| Hepadnaviridae | 3 |
| Endornaviridae | 3 |
| Closteroviridae | 2 |
| Leviviridae | 2 |
| Circoviridae | 2 |
| Luteoviridae | 1 |
| Potyviridae | 1 |
| Picobirnaviridae | 1 |

**Table S3:** Alignment of the sequences of the different aichi viruses.

|  | | | | | | | | | | | | | | | | | | | | | | | | | | | | | | |
| --- | --- | --- | --- | --- | --- | --- | --- | --- | --- | --- | --- | --- | --- | --- | --- | --- | --- | --- | --- | --- | --- | --- | --- | --- | --- | --- | --- | --- | --- | --- |
| Sample 1 | C | C | C | C | G | A | T | C | A | T | G | T | C | G | T | A | C | A | T | C | T | G | G | T | T | G | C | C | A | T |
| Sample 2 | . | . | . | . | . | . | . | . | . | . | . | . | . | . | . | . | . | . | . | G | . | . | . | . | . | . | . | . | . | . |
| Sample 3 | . | . | . | . | . | . | . | . | . | . | . | . | . | . | . | . | . | . | . | . | . | . | . | . | . | . | . | . | . | . |
| Sample 4 | . | . | . | . | . | . | . | . | . | . | . | . | . | . | . | . | . | . | . | . | . | . | A | . | . | . | . | . | . | . |
| Sample 5 | . | . | . | . | . | . | . | . | . | . | . | . | . | . | . | . | . | . | . | . | . | . | . | . | . | . | . | . | . | . |
| Sample 6 | . | . | . | . | . | . | . | . | . | . | . | . | . | . | . | . | . | . | . | . | C | . | . | . | . | . | . | . | . | . |
| Sample 7 | . | . | . | . | . | . | . | . | . | . | . | . | . | . | . | . | . | . | . | . | . | . | . | . | . | . | . | . | . | . |
| Sample 8 | . | . | . | . | . | . | . | . | . | . | . | . | . | . | . | . | . | . | . | . | . | . | . | . | . | . | . | . | . | . |
| Sample 9 | . | . | . | . | . | . | . | . | . | . | . | . | . | . | . | . | . | . | . | . | . | . | . | . | . | . | . | . | . | . |
| Sample 10 | . | . | . | . | . | . | . | . | . | . | . | . | . | . | . | . | . | . | . | . | . | . | . | . | . | . | . | . | . | . |
| Sample 11 | . | . | . | . | . | . | . | . | . | . | . | . | . | . | . | . | . | . | . | . | C | . | . | . | . | . | . | . | . | . |
| Sample 12 | . | . | . | . | . | . | . | . | . | . | . | . | . | . | . | . | . | . | . | . | . | . | A | . | . | . | . | . | . | . |
| Sample 13 | . | . | . | . | . | . | . | . | . | . | . | . | . | . | . | . | . | . | . | . | C | . | . | . | . | . | . | . | . | . |
| Sample 14 | . | . | . | . | . | . | . | . | . | . | . | . | . | . | . | . | . | . | . | G | . | . | . | . | . | . | . | . | . | . |
| Sample 15 | . | . | . | . | . | . | . | . | . | . | . | . | . | . | . | . | . | . | . | . | C | . | . | . | . | . | . | . | . | . |
| Sample 16 | . | . | . | . | . | . | . | . | . | . | . | . | . | . | . | . | . | . | . | . | C | . | . | . | . | . | . | . | . | . |
| Sample 17 | . | . | . | . | . | . | . | . | . | . | . | . | . | . | . | . | . | . | . | . | . | . | A | . | . | . | . | . | . | . |
| Sample 18 | . | . | . | . | . | . | . | . | . | . | . | . | . | . | . | . | . | . | . | . | C | . | A | . | . | . | . | . | . | . |
| Sample 19 | . | . | . | . | . | . | . | . | . | . | . | . | . | . | . | . | . | . | . | G | . | . | . | . | . | . | . | . | . | . |
| Sample 20 | . | . | . | . | . | . | . | . | . | . | . | . | . | . | . | . | . | . | . | . | . | . | . | . | . | . | . | . | . | . |
| Sample 21 | . | . | . | . | . | . | . | . | . | . | . | . | . | . | . | . | . | . | . | . | C | . | . | . | . | . | . | . | . | . |
| Sample 22 | . | . | . | . | . | . | . | . | . | . | . | . | . | . | . | . | . | . | . | . | . | . | . | . | . | . | . | . | . | . |
| Sample 23 | . | . | . | . | . | . | . | . | . | . | . | . | . | . | . | . | . | . | . | . | . | . | . | . | . | . | . | . | . | . |
| Sample 24 | . | . | . | . | . | . | . | . | . | . | . | . | . | . | . | . | . | . | . | . | C | . | . | . | . | . | . | . | . | . |
| Sample 25 | . | . | . | . | . | . | . | . | . | . | . | . | . | . | . | . | . | . | . | . | . | . | . | . | . | . | . | . | . | . |
| Sample 26 | . | . | . | . | . | . | . | . | . | . | . | . | . | . | . | . | . | . | . | . | . | . | . | . | . | . | . | . | . | . |
| Sample 27 | . | . | . | . | . | . | . | . | . | . | . | . | . | . | . | . | . | . | . | . | . | . | . | . | . | . | . | . | . | . |
| Sample 28 | . | . | . | . | . | . | . | . | . | . | . | . | . | . | . | . | . | . | . | . | . | . | . | . | . | . | . | . | . | . |
| Sample 29 | . | . | . | . | . | . | . | . | . | . | . | . | . | . | . | . | . | . | . | . | . | . | . | . | . | . | . | . | . | . |
| Sample 30 | . | . | . | . | . | . | . | . | . | . | . | . | . | . | . | . | . | . | . | . | C | . | . | . | . | . | . | . | . | . |
| Sample 31 | . | . | . | . | . | . | . | . | . | . | . | . | . | . | . | . | . | . | . | . | C | . | . | . | . | . | . | . | . | . |
| Sample 32 | . | . | . | . | . | . | . | . | . | . | . | . | . | . | . | . | . | . | . | . | C | . | . | . | . | . | . | . | . | . |
| Sample 33 | . | . | . | . | . | . | . | . | . | . | . | . | . | . | . | . | . | . | . | . | . | . | . | . | . | . | . | . | . | . |
| Sample 34 | . | . | . | . | . | . | . | . | . | . | . | . | . | . | . | . | . | . | . | . | . | . | A | . | . | . | . | . | . | . |
| Sample 35 | . | . | . | . | . | . | . | . | . | . | . | . | . | . | . | . | . | . | . | . | . | . | . | . | . | . | . | . | . | . |
| Sample 36 | . | . | . | . | . | . | . | . | . | . | . | . | . | . | . | . | . | . | . | . | . | . | . | . | . | . | . | . | . | . |
| Sample 37 | . | . | . | . | . | . | . | . | . | . | . | . | . | . | . | . | . | . | . | . | . | . | . | . | . | . | . | . | . | . |
| Sample 38 | . | . | . | . | . | . | . | . | . | . | . | . | . | . | . | . | . | . | . | . | . | C | . | . | . | . | . | . | . | . |
| Sample 39 | - | - | - | - | - | - | - | - | . | . | . | . | . | . | . | . | . | . | . | . | . | . | n | . | . | . | . | . | . | . |
| Sample 40 | - | - | - | - | - | - | - | - | - | - | - | - | - | - | - | . | . | . | . | . | C | . | . | . | . | . | . | . | . | . |
|  |  |  |  |  |  |  |  |  |  |  |  |  |  |  |  |  |  |  |  |  |  |  |  |  |  |  |  |  |  |  |
| Sample 1 | A | G | A | T | G | T | G | A | T | G | T | G | A | A | T | G | G | C | G |  |  |  |  |  |  |  |  |  |  |  |
| Sample 2 | . | . | . | . | . | . | . | . | . | . | . | . | . | . | . | . | . | . | . |  |  |  |  |  |  |  |  |  |  |  |
| Sample 3 | . | . | . | . | . | . | . | G | . | . | . | . | . | . | . | . | . | . | . |  |  |  |  |  |  |  |  |  |  |  |
| Sample 4 | . | . | . | . | . | . | . | G | . | . | . | . | . | . | . | . | . | . | . |  |  |  |  |  |  |  |  |  |  |  |
| Sample 5 | . | . | . | . | . | . | . | G | . | . | . | . | . | . | . | . | . | . | . |  |  |  |  |  |  |  |  |  |  |  |
| Sample 6 | . | . | . | . | . | . | . | G | . | . | . | . | . | . | . | . | . | . | . |  |  |  |  |  |  |  |  |  |  |  |
| Sample 7 | . | . | . | . | . | . | . | G | . | . | . | . | . | . | . | . | . | . | . |  |  |  |  |  |  |  |  |  |  |  |
| Sample 8 | . | . | . | . | . | . | . | G | . | . | . | . | . | . | . | . | . | . | . |  |  |  |  |  |  |  |  |  |  |  |
| Sample 9 | . | . | . | . | . | . | . | G | . | . | . | . | . | . | . | . | . | . | . |  |  |  |  |  |  |  |  |  |  |  |
| Sample 10 | . | . | . | . | . | . | . | G | . | . | . | . | . | . | . | . | . | . | . |  |  |  |  |  |  |  |  |  |  |  |
| Sample 11 | . | . | . | . | . | . | . | G | . | . | . | . | . | . | . | . | . | . | . |  |  |  |  |  |  |  |  |  |  |  |
| Sample 12 | . | . | . | . | . | . | . | . | . | . | . | . | . | . | . | . | . | . | . |  |  |  |  |  |  |  |  |  |  |  |
| Sample 13 | . | . | . | . | . | . | . | G | . | . | . | . | . | . | . | . | . | . | . |  |  |  |  |  |  |  |  |  |  |  |
| Sample 14 | . | . | . | . | . | . | . | G | . | . | . | . | . | . | . | . | . | . | . |  |  |  |  |  |  |  |  |  |  |  |
| Sample 15 | . | . | . | . | . | . | . | G | . | . | . | . | . | . | . | . | . | . | . |  |  |  |  |  |  |  |  |  |  |  |
| Sample 16 | . | . | . | . | . | . | . | G | . | . | . | . | . | . | . | . | . | . | . |  |  |  |  |  |  |  |  |  |  |  |
| Sample 17 | . | . | . | . | . | . | . | . | . | . | . | . | . | . | . | . | . | . | . |  |  |  |  |  |  |  |  |  |  |  |
| Sample 18 | . | . | . | . | . | . | . | G | . | . | . | . | . | . | . | . | . | . | . |  |  |  |  |  |  |  |  |  |  |  |
| Sample 19 | . | . | . | . | . | . | . | . | . | . | . | . | . | . | . | . | . | . | . |  |  |  |  |  |  |  |  |  |  |  |
| Sample 20 | . | . | . | . | . | . | . | G | . | . | G | . | . | . | . | . | . | . | . |  |  |  |  |  |  |  |  |  |  |  |
| Sample 21 | . | . | . | . | . | . | . | G | . | . | . | . | . | . | . | . | . | . | . |  |  |  |  |  |  |  |  |  |  |  |
| Sample 22 | . | . | . | . | . | . | . | G | . | . | A | . | . | . | . | . | . | . | . |  |  |  |  |  |  |  |  |  |  |  |
| Sample 23 | . | . | . | . | . | . | . | G | . | . | . | . | . | . | . | . | . | . | . |  |  |  |  |  |  |  |  |  |  |  |
| Sample 24 | . | . | . | . | . | . | . | G | . | . | . | . | . | . | . | . | . | . | . |  |  |  |  |  |  |  |  |  |  |  |
| Sample 25 | . | . | . | . | . | . | . | G | . | . | G | . | . | . | . | . | . | . | . |  |  |  |  |  |  |  |  |  |  |  |
| Sample 26 | . | . | . | . | . | . | . | G | . | . | G | . | . | . | . | . | . | . | . |  |  |  |  |  |  |  |  |  |  |  |
| Sample 27 | . | . | . | . | . | . | . | G | . | . | G | . | . | . | . | . | . | . | . |  |  |  |  |  |  |  |  |  |  |  |
| Sample 28 | . | . | . | . | . | . | . | G | . | . | G | . | . | . | . | . | . | . | . |  |  |  |  |  |  |  |  |  |  |  |
| Sample 29 | . | . | . | . | . | . | . | G | . | . | G | . | . | . | . | . | . | . | . |  |  |  |  |  |  |  |  |  |  |  |
| Sample 30 | . | . | . | . | . | . | . | G | . | . | . | . | . | . | . | . | . | . | . |  |  |  |  |  |  |  |  |  |  |  |
| Sample 31 | . | . | . | . | . | . | . | G | . | . | . | . | . | . | . | . | . | . | . |  |  |  |  |  |  |  |  |  |  |  |
| Sample 32 | . | . | . | . | . | . | . | G | . | . | . | . | . | . | . | . | . | . | . |  |  |  |  |  |  |  |  |  |  |  |
| Sample 33 | . | . | . | . | . | . | . | G | . | . | G | . | . | . | . | . | . | . | . |  |  |  |  |  |  |  |  |  |  |  |
| Sample 34 | . | . | . | . | . | . | . | G | . | . | G | . | . | . | . | . | . | . | . |  |  |  |  |  |  |  |  |  |  |  |
| Sample 35 | . | . | . | . | . | . | . | G | . | . | G | . | . | . | . | . | . | . | . |  |  |  |  |  |  |  |  |  |  |  |
| Sample 36 | . | . | . | . | . | . | . | . | . | . | . | . | . | . | . | . | . | . | . |  |  |  |  |  |  |  |  |  |  |  |
| Sample 37 | . | . | . | . | . | . | . | G | . | . | . | . | . | . | . | . | . | . | . |  |  |  |  |  |  |  |  |  |  |  |
| Sample 38 | . | . | . | . | . | . | . | G | . | . | G | . | . | . | . | . | . | . | . |  |  |  |  |  |  |  |  |  |  |  |
| Sample 39 | . | n | . | . | . | . | . | G | . | . | . | . | . | . | . | . | . | . | . |  |  |  |  |  |  |  |  |  |  |  |
| Sample 40 | . | . | . | . | . | . | . | G | . | . | . | . | . | . | . | . | . | . | . |  |  |  |  |  |  |  |  |  |  |  |

**Figure S1: Pipeline of the data analysis**


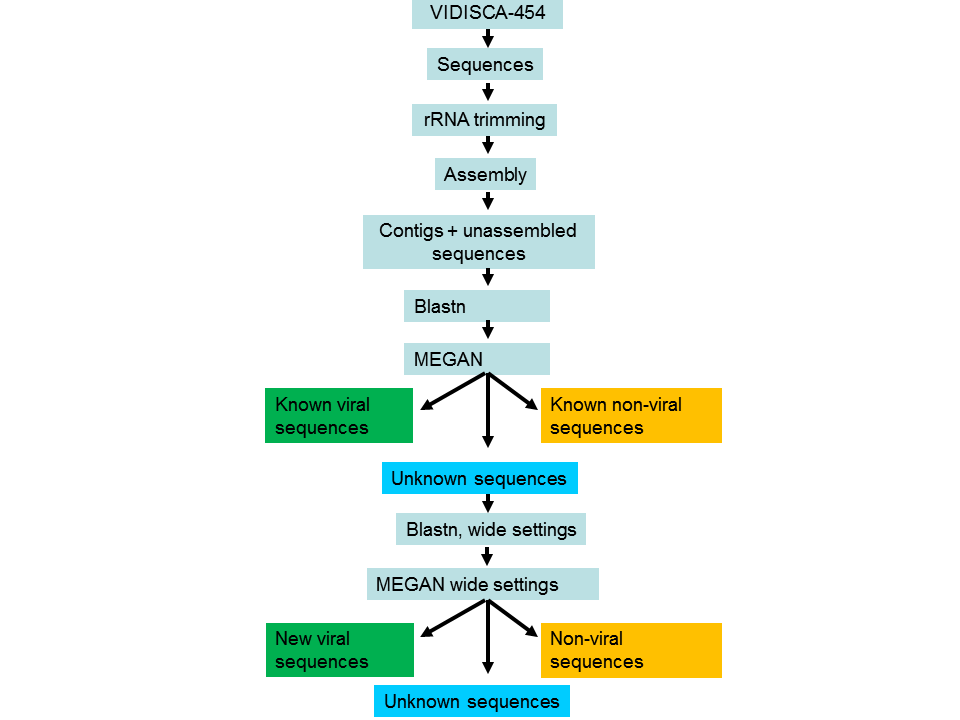

Supplement: Additional file 1: Table S1 — Primer combination used for the screening. Table S2: Quantification of the detection of viral families in stool samples of HIV-1 infected individuals. Table S3: Alignment of the sequences of the different aichi viruses. Figure S1: Pipeline of the data analysis. [file 1471-2334-14-22-S1.doc]
